# The Effect of Subgingival Curcumin Gel With and Without Photodynamic Therapy as Adjunctive Periodontal Treatment in Type 2 Diabetes Mellitus Patients: A Split-Mouth Clinical and Microbiological Study

**DOI:** 10.1155/ijod/2575672

**Published:** 2025-03-07

**Authors:** Doosadee Hormdee, Nisachol Tarawadee, Weena Rinsathorn, Subin Puasiri, Waraporn Suwannarong

**Affiliations:** ^1^Division of Periodontology, Department of Oral Biomedical Sciences, Faculty of Dentistry and Research Group of Chronic Inflammatory Oral Diseases and Systemic Diseases Associated with Oral Health, Khon Kaen University, Khon Kaen 40002, Thailand; ^2^Division of Periodontology, Department of Oral Biomedical Sciences, Faculty of Dentistry, Khon Kaen University, Khon Kaen, Thailand; ^3^Division of Community Dentistry, Department of Oral Prevention, Faculty of Dentistry, Khon Kaen University, Khon Kaen 40002, Thailand

**Keywords:** *Curcuma longa*, diabetes mellitus type 2, microbiology, periodontal diseases, photochemotherapy

## Abstract

**Background:** The study aimed to determine the quantity of periodontopathic and total bacteria on subgingival plaque from patients with periodontitis and uncontrolled diabetes and to compare adjunctive periodontal therapy using topical *Curcuma longa* extract gel with and without photodynamic treatment using blue light-emitting diodes (LEDs). Clinical periodontal parameters such as probing depth (PD), clinical attachment level (CAL), and bleeding on probing (BOP) were assessed to evaluate the efficacy of the treatments.

**Methods:** Thirty patients with diabetes mellitus (DM), a history of poor glycemic control, and chronic periodontitis were enrolled in this clinical and microbiological split-mouth study. After conventional periodontal treatment, scaling, and root planing, the most severe sites on the premolar or molar teeth with periodontal pockets measuring ≥5 mm were selected and randomly divided into two groups: the *C. longa* extract gel group (with a concentration of 25 µg/g) and the photodynamic group (*C. longa* extract gel 25 µg/g irradiated with blue light energy density = 16.8 J/cm^2^ for 120 s). All teeth were examined for clinical periodontal parameters data (PD, CAL, plaque index [PI], and BOP) and microbiological data (*Porphyromonas gingivalis*, *Prevotella intermedia*, *Fusobacterium nucleatum*, *Aggregatibacter actinomycetemcomitans*, and total bacteria), measured using TaqMan Multiplex real-time polymerase chain reaction, at weeks 0, 1, 2, and 12. The Mann–Whitney *U* test was utilized to compare clinical outcomes between groups, while the Wilcoxon Signed Rank test at a 95% confidence level was employed to analyze the amount of periodontal pathogens within the same group.

**Results:** After the 12-week follow-up period, significant improvements were observed in all clinical parameters across both groups. However, the reduction in both CAL and BOP was significantly higher in the photodynamic group compared to the curcumin gel alone group, indicating enhanced periodontal health outcomes in the former. Microbiologically, both groups exhibited a decrease in total bacterial count and a reduction in periodontopathic bacteria. Importantly, the photodynamic group demonstrated a significant decrease in *F. nucleatum* and *P. intermedia* counts, key pathogens associated with periodontal disease progression. This suggests that photodynamic therapy (PDT), when used with curcumin gel, not only improves clinical parameters but also promotes a favorable shift in the periodontal microbial profile.

**Conclusion:** Our findings highlight that PDT with curcumin gel as a photosensitizer (PS) is more effective than curcumin gel alone in achieving clinical attachment gain, reducing gingival inflammation, and suppressing specific periodontal pathogens. This combined therapy approach holds promise for managing periodontal disease by addressing both clinical symptoms and microbial factors.

## 1. Introduction

Diabetes mellitus (DM) is a group of metabolic disorders characterized by abnormally high blood glucose levels (hyperglycemia) [1]. Periodontitis is a common oral disease characterized by dysbiosis of the biofilm, which leads to the destruction of the tooth-supporting structures, resulting in tooth loss and contributing to other health problems [2–4]. Many pieces of evidence support the existence of a two-way relationship between diabetes and periodontitis; both can increase and exacerbate systemic inflammation [5–7].

Conventional periodontal therapy, scaling and root planing (SRP), significantly reduce the number of bacteria by eliminating dental plaque and calculus. However, this method in the treatment of periodontal diseases still has limitations when used in some areas of the teeth and has no further bactericidal or anti-inflammation effects [8, 9]. Currently, adjunctive periodontal treatment, including local antibiotics, antiseptics, and herbal products, such as *Curcuma longa*, has been used to address these limitations [10–13]. Additionally, advancements in periodontal therapy have led to the exploration of probiotics, paraprobiotics, and postbiotics as potential adjunctive treatments. These natural agents offer promising avenues for modulating the oral microbiome and reducing the bacterial load associated with periodontal disease, thereby supporting periodontal health and enhancing treatment outcomes [14–17].

Patients with poorly controlled diabetes were specifically selected for this study due to their heightened susceptibility to periodontal complications, which conventional therapies may not adequately address [18–21]. DM patients are known to be more susceptible to increased bacterial recolonization in the gingival sulcus compared to healthy individuals [22–24]. The synergistic effects of *C. longa* extract gel and photodynamic therapy (PDT), as demonstrated in our previous in vitro and clinical studies [25, 26], are expected to provide superior clinical outcomes. PDT involves using blue light-emitting diodes (LEDs) to activate the antibacterial properties of *C. longa* extract gel, potentially enhancing its efficacy in reducing periodontal pathogens compared to using the gel alone. Therefore, this study aims to investigate the effectiveness of the subgingival application of *C. longa* extract gel along with blue LED light irradiation compared with the application of the gel alone as an adjunct to conventional SRP procedures in patients suffering from periodontitis and poorly controlled glycemic levels.

## 2. Materials and Methods

### 2.1. Study Design and Participants

Thirty patients with a history of uncontrolled DM and generalized periodontitis (stages III–IV, grade C) were enrolled in the study after the study purpose was explained and their written consent was obtained. The inclusion criteria were diabetes patients aged between 45 and 65 years old who had HbA1c of more than 7.0% and no periodontal treatment 3 months before the study. Patients with systemic diseases that could influence the outcomes, systemic antibiotic, or steroid use within 3 months before the study, pregnancy or lactating mothers, smoking or betel chewing, and allergy to turmeric were excluded. The sample size for this study was calculated based on methodologies from similar clinical studies by Hormdee et al. [25] and Chondros et al. [27], setting *α* at 0.05 and statistical power at 0.80.

### 2.2. Clinical Examination and Treatment

All clinical procedures, except for group randomization and PDT administration, were performed by a single examiner with over 3 years of specialized training in periodontology. The examiner underwent rigorous calibration exercises, and intra-examiner reliability was validated before the study commenced, with interclass correlation coefficients (ICCs) exceeding 0.9. Periodontal examination was conducted before active periodontal treatment (baseline) and after an appropriate healing period (12 weeks) [28].

The treatment began with full-mouth SRP, conducted by the primary dentist using a piezoelectric dental scaler (Acteon Newtron P5 XS, France) and hand instruments (Gracey Curettes, Hu-Friedy, Chicago, IL, USA). Patients were also given oral hygiene instructions. Following SRP, premolar or molar teeth with periodontal pockets ≥5 mm were randomly assigned into two groups by the main researcher, ensuring treatment allocation remained undisclosed to others. The control group, the Gel group, received only 25 µg/g *C. longa* extract gel (developed by the Center for Research and Development of Herbal Health Products, Thailand, according to good manufacturing practice standards) and the experimental group, the Gel + PDT group, received the same gel, followed by irradiation with 420–480 nm wavelength blue LED light at an energy density of 16.8 J/cm^2^ for 120 s. After SRP and saline irrigation, *C. longa* gel was gently applied into the periodontal pockets until extrusion, with excess gel removed using cotton rolls. For the gel + PDT group, photodynamic therapy was performed by applying the blue LED (curing light—Mini LED Supercharged, Acteon, Norwich, England), connected to a quartz tip, periodontal probe-like in shape with a round endpoint of 1 mm in diameter. The LED was placed at the depth of the pocket and moved circumferentially around the tooth for 120 s to ensure uniform irradiation of the affected area. Patients were instructed not to rinse, drink, or eat for 1 h after gel application and to avoid using any other mouthwash. In the experimental group, the irradiation mentioned earlier was conducted by the main researcher.

The following clinical parameters: plaque index (PI), periodontal probing depth (PD), clinical attachment level (CAL), and bleeding on probing (BOP), six sites per tooth were measured and recorded at weeks 0 and 12. The PI was reported as scores of 0–3 according to The Silness and Löe [29] and Löe Plaque Index. PD was measured to the nearest millimeter using a standard periodontal probe (UNC 15, Hu-Friedy, Chicago, IL, USA) with a trained probing force of ≈0.2–0.3 N and represents the distance from the bottom of the pocket to the gingival margin, as proposed by Gabathuler and Hassell [30]. CAL was recorded as the distance from the bottom of the periodontal pocket to the cemento-enamel junction (CEJ). BOP was recorded as “present” or “absent” after probing, according to Van der Weijden et al. [31]. All adjunctive procedures were repeated three times at weekly intervals. At a 3-month follow-up appointment, the primary dentist examined and recorded periodontal parameters.

### 2.3. Microbial Samples

Subgingival plaque samples were collected from treated sites at weeks 0, 1, 2, and 12. The supragingival plaque was removed by sterile cotton pellets. The tooth site was dried and isolated from the saliva by cotton rolls. Three sterile paper points per site (Dentsply Sirona, North Carolina, United States) were inserted into the periodontal pocket to collect subgingival plaque. All three paper points were pooled and then immediately suspended in 150 µL of RNAlater storage solution (InvitrogenTM, Massachusetts, United States) and stored at −20°C.

The microbiological data were measured using TaqMan Multiplex real-time polymerase chain reaction (Thermo Scientific, Massachusetts, United States) at weeks 0, 1, 2, and 12. The bacterial DNA was extracted from clinical plaque samples using a GeneJET Genomic DNA Purification Kit (Thermo Scientific) according to the manufacturer's instructions. The purified DNA was then used for TaqMan Multiplex real-time polymerase chain reaction to quantify *Porphyromonas gingivalis*, *Prevotella intermedia*, *Fusobacterium nucleatum*, *Aggregatibacter actinomycetemcomitans*, and 16s rDNA as total bacteria. The primers and probes used are shown in Table 1. *P. gingivalis* ATCC33277, *P. intermedia* ATCC25611, *A. actinomycetemcomitans* ATCC43718 and *F. nucleatum* ATCC10953 were used as references for periodontopathic bacteria. Then *Streptococcus mutans* ATCC25175, *Streptococcus sanguinis* ATCC10556, *Staphylococcus aureus* ATCC 25923, and *Lactobacillus acidophilus* ATCC 4356 were used as references for total bacteria. The amplification conditions for quantitative real-time PCR used in this study consisted of an initial denaturation stage at 95°C for 10 min, followed by 35 cycles of denaturation at 95°C for 15 s, annealing, and extension at 58°C for 1 min. A standard curve was used to estimate the DNA concentration of unknown samples by comparing them to standards with known DNA concentrations.

### 2.4. Data Analysis

The normality of the data was assessed using the Kolmogorov–Smirnov test. Clinical outcomes between groups at weeks 0 and 12 were compared using the Mann–Whitney *U* test for data that were not normally distributed. Within-group comparisons before and after treatment were analyzed using the Wilcoxon signed-rank test, with significance set at *p*  < 0.05. Additionally, data comparing the amount of periodontal pathogens within the same group were analyzed using a median model with the Wilcoxon Signed Rank test at a 95% confidence level. Data were analyzed by SPSS statistics for Windows, Version 21.0 (SPSS Inc., Chicago, United States).

## 3. Results

**Figure 1:**
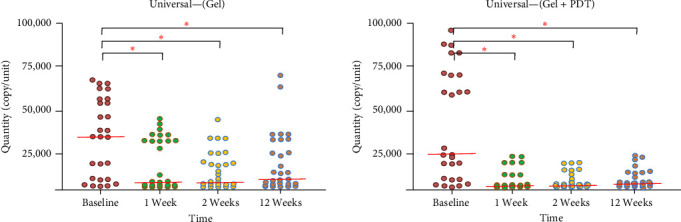
Median and quantity of total balcteria from baseline through 12 weeks follow-up of the study groups. *⁣*^*∗*^Indicate statistical significance levels determined by the Wilcoxon signed-rank test, with a significance threshold set at *p* < 0.05. Colored circles represent individual samples from one site of the defect.

**Figure 2:**
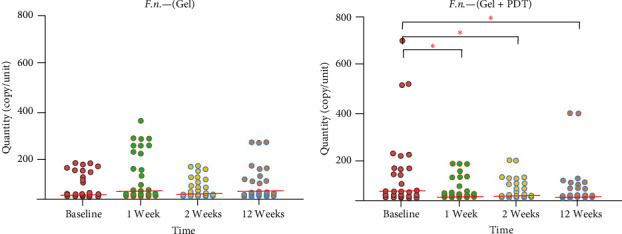
Median and quantity of *F. nucleatum* from baseline through 12 weeks follow-up of the study groups. *⁣*^*∗*^Indicate statistical significance levels determined by the Wilcoxon signed-rank test, with a significance threshold set at *p* < 0.05. Colored circles represent individual samples from one site of the defect.

**Figure 3:**
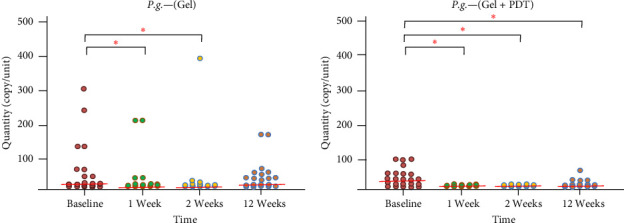
Median and quantity of *P. gingivalis* from baseline through 12 weeks follow-up of the study groups. *⁣*^*∗*^Indicate statistical significance levels determined by the Wilcoxon signed-rank test, with a significance threshold set at *p* < 0.05. Colored circles represent individual samples from one site of the defect.

**Figure 4:**
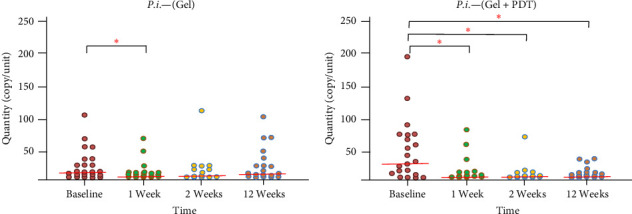
Median and quantity of *P. intermedia* from baseline through 12 weeks follow-up of the study groups. *⁣*^*∗*^Indicate statistical significance levels determined by the Wilcoxon signed-rank test, with a significance threshold set at *p* < 0.05. Colored circles represent individual samples from one site of the defect.

**Figure 5:**
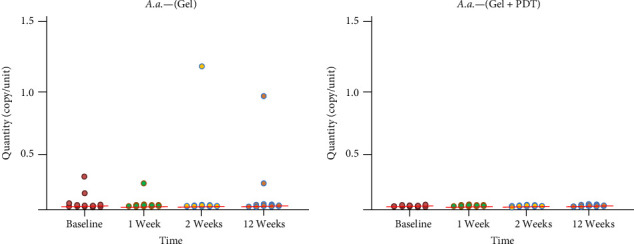
Median and Quantity of *A. actinomycetemcomitans* from baseline through 12 weeks follow up of the study groups. Wilcoxon signed-rank test was employed with a significance level set at *p*  < 0.05. Colored circles represent individual samples from one site of the defect.

Thirty patients were included in the study. Three patients were excluded during the follow-up: one patient did not complete the follow-up, and two were excluded due to receiving systemic antibiotic therapy for other infections. Therefore, a total of 27 patients, including 14 males and 13 females aged between 47 and 64 years old, were included in the study. No complications or adverse effects from the adjunctive treatment were reported during the treatments. The microbiological data presented in Figure 1 indicate that total bacteria in the periodontal pocket were significantly reduced at weeks 1, 2, and 12 after adjunctive treatment in both the Gel and Gel + PDT groups. In the Gel + PDT group, reductions in *F. nucleatum*, *Porphyromonas gingivalis*, and *Prevotella intermedia* (Figures 2–4) were significantly different from baseline throughout the 12-week follow-up period. Conversely, in the Gel group, reductions in *Porphyromonas gingivalis* and *Prevotella intermedia* (Figures 3 and 4) were significantly different only at weeks 1 and 2 posttreatment. This study found no significant differences in *Aggregatibacter actinomycetemcomitans* (Figure 5) among the study groups. All statistical significance was determined using the Wilcoxon signed-rank test with a significance level set at *p* < 0.05. However, the intergroup comparison revealed that only *F. nucleatum* and *Prevotella intermedia* were significantly reduced in the Gel + PDT group compared with the Gel group.

### 3.1. Clinical and Microbiological Outcomes

The median and interquartile range of HbA1c levels for all patients are presented in Table 2. After conservative and adjunctive periodontal treatments, there was a decrease in the HbA1c percentage from 10.0 to 9.1 (equivalent to 86–76 mmol/mol) over the 3-month follow-up period when patients were subgrouped based on their HbA1c levels (Table 2), with “high” defined as HbA1c 6.5%–8.0% and “extremely high” as HbA1c 8.1%–11%, only half of those with high HbA1c levels demonstrated a decrease, while 13 out of 21 patients with extremely high HbA1c levels experienced a reduction. Remarkably, the mean reduction in the extremely high group was twice that in the high group: 0.74% compared to 0.32%, respectively.

At baseline, no significant differences between the study groups were found in the oral clinical parameters (PD, CAL, PI, and BOP). After the adjunctive treatment, all clinical parameters significantly improved (*p*  < 0.001). Furthermore, the intergroup comparison showed a statistically significant reduction in CAL and BOP in the Gel + PDT group compared with the Gel group at week 12 (Table 3).

Microbiological data presented in Figure 1 indicate that total bacteria in the periodontal pocket were significantly reduced at weeks 1, 2, and 12 after adjunctive treatment in both the Gel and the Gel + PDT groups. In the Gel + PDT group, reductions in *F.n*., *P.g*., *and P.i*. (Figures 2–4) were significantly different from baseline throughout the 12-week follow-up period. Conversely, in the Gel group, reductions in *P.g*. and *P.i*. (Figures 3–4) were significantly different only at weeks 1 and 2 post-treatments. This study found no significant differences in *A.a*. (Figure 5) among the study groups. However, the intergroup comparison revealed that only *F.n*. and *P.i*. were significantly reduced in the Gel + PDT group when compared with the Gel group.

**Figure 6:**
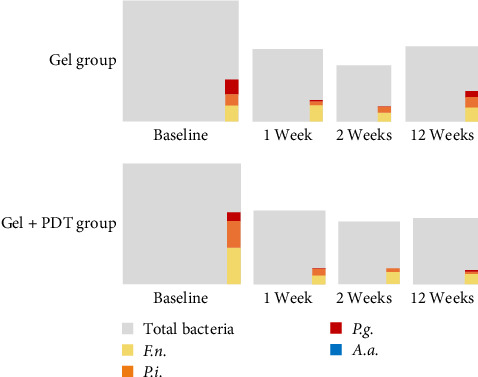
The ratio of periodontopathic bacteria to universal bacteria from baseline through 12 weeks follow-up of the study groups.

The number of bacteria in the periodontal pocket, obtained through TaqMan Multiplex real-time polymerase chain reaction, was utilized to generate images (Figure 6) illustrating the ratio of periodontopathic bacteria to total bacteria. Despite the reduction in total bacteria following the treatments, both groups showed a slight increase in total bacterial recolonization at week 12 compared to week 2 posttreatment. The reduction in the four periodontopathic bacteria was evident in both groups following treatment. Additionally, the Gel + PDT group demonstrated a persistent effect on antibacterial recolonization from week 1 through week 12 of follow-up.

## 4. Discussion

There is strong evidence that periodontitis is associated with diabetes. Furthermore, diabetes complications and glycemic control are related to periodontitis in a dose-response manner [33]. Recently published meta-analyses showed a modest (0.56% CI 0.36–0.75) but significant reduction in HbA1c following periodontal therapy [34]. Our results show a lower reduction in the patient who has HbA1c <8% at baseline but can show a higher reduction in the extremely high HbA1c level group (0.74% CI 0.69–0.87). Additionally, administering local curcumin gel in addition to SRP in patients with uncontrolled diabetes achieved a reduction in glycemic control.

In recent decades, numerous studies have highlighted the potential of curcumin and its extracts in treating periodontitis. Various forms of curcuminoid products—such as solutions, chips, gels, capsules, and combinations with other periodontal treatments like PDT—have been explored [35–38]. To the best of our knowledge, no study has reported comparing multiple shots of using curcumin gel as photosensitizer (PS) and as local subgingival adjunctive treatment after SRP in patients suffering from uncontrol diabetes. Overall, our clinical results demonstrated statistically significant improvements in PD, CAL, BOP, and PI following periodontal treatment in both groups. Moreover, using curcumin gel as a PS in photodynamic therapy showed significant benefits in CAL and BOP compared to its use solely as a local subgingival adjunctive treatment. As previously reported, the production of hydroxyl radicals and the antirecolonization effects of curcumin gel when irradiated with specific wavelength light may not only explain our in vitro findings but also contribute to the observed clinical improvements in these poorly controlled diabetic patients.

Regarding clinical trials, few studies have evaluated PDT as an adjunctive therapy in chronic periodontitis patients with type 2 DM undergoing nonsurgical treatment. Studies employing single-session PDT did not show additional benefits in PD and CAL parameters [39–41]. Due to differences in the study protocols, performing PDT once a week for 3 consecutive weeks was able to retrieve better clinical results and recolonization of periodontal bacteria. Hence, multiple subgingival applications must be considered for these sensitive patients.

Several studies have revealed that DM contributes to the pathophysiological mechanism for microbial dysbiotic changes during periodontitis progression [42–46]. In current study showed a dramatic reduction of total bacteria and periodontal pathogens followed by using curcumin gel form in the subgingival sulcus and irradiated with light 420–480 nm wavelength, 16.8 J/cm^2^ for 120 s. At the 12 weeks after treatment, the number of Gram-negative bacteria and the ratio of periodontopathic bacteria to newly colonized total bacteria were almost doubled in the curcumin gel group but not in the PDT group. This might be due to the curcumin-PS attached to the bacterial cell wall of periodontal pathogens, and when irradiated with a specific wavelength, light produces reactive oxygen species (ROS), which can destroy the pathogen in biofilm and gingival sulcus [25, 26]. While our study demonstrated statistically significant clinical improvement, it is important to acknowledge the significance of considering other recently introduced compounds that have been shown to influence the oral environment [15–17]. However, a limitation of our research lies in its focus only on curcumin, neglecting the potential synergistic effects of other emerging compounds such as postbiotics, lysates, and pParaprobiotics, which may also influence clinical parameters in periodontal patients. Therefore, future research reports are needed to explore these emerging compounds and their potential synergistic effects, and additional studies with larger sample sizes and longer durations are necessary to provide further confirmation of the effects of this PDT. Furthermore, integrating the use of ozonated substances to reduce the incidence of bacterial load, along with utilizing the antioxidant action of photodynamic therapy, should be considered as important future objectives to enhance periodontal treatment approaches.

## 5. Conclusions

In conclusion, both adjunctive periodontal therapies have a treatment influence on the periodontal pocket in a short-term study (12 weeks). *C. longa* extract gel as a PS in photodynamic therapy is more effective than *Curcuma Longa* extract gel alone in clinical attachment gain, attenuated gingival inflammation, and reduced *F. nucleatum and P. intermedia* in the periodontal pocket. Moving forward, proactive action is needed to further investigate the long-term efficacy and optimize the implementation of these adjunctive therapies for improved periodontal treatment outcomes.

## Figures and Tables

**Table 1:** Target primers/probe sequence for quantitative real-time PCR used in this study [32].

Bacteria	Primer sequence (5′-3′)
*A. actinomycetemcomitans*	Forward: CTT ACC TAC TCT TGA CAT CCG AA Reverse: ATG CAG CAC CTG TCT CAA AGC Probe: JUN-AGA ACT CAG AGA TGG GTT TGT GCC TTA G-QSY

*P. gingivalis*	Forward: TGC AAC TTG CCT TAC AGA GGG Reverse: ACT CGT ATC GCC CGT TAT TC Probe: FAM-AGC TGT AAG ATA GGC ATG CGT CCC ATT AGC TA-QSY

*P. intermedia*	Forward: CCA CAT ATG GCA TCT GAC GTG Reverse: TCA ATC TGC ACG CTA CTT GG Probe: FAM-ACC AAA GAT TCA TCG GTG GAG GAT GGG-QSY

*F. nucleatum*	Forward: CGC AGA AGG TGA AAG TCC TGT AT Reverse: TGG TCC TCA CTG ATT CAC ACA GA Probe: ABY-ACT TTG CTC CCA AGT AAC ATG GAA CAC GAG-QSY

16s rDNA (total bacteria)	Forward: TCC TAC GGG AGG CAG CAGT Reverse: GGA CTA CCA GGG TAT CTA ATC CTG TT Probe: ABY-CGT ATT ACC GCG GCT GCT GGC AC-QSY

**Table 2:** HbA1c data of patients.

(A)		
HbA1c level	Before treatment (*n* = 27)	After treatment (*n* = 27)
Median (Q1, Q3)	10.0% (8.2, 10.5)	9.1% (8.0, 10.2)

**(B)**		
	**High (*n* = 6) HbA1c 6.5%–8.0%**	**Extremely high (*n* = 21) HbA1c 8.1%–11.0%**

Cases of HbA1c reduction	3 out of 6	13 out of 21
Mean *Δ*HbA1c	0.32 %	0.74%

**Table 3:** Mean, standard deviation, and median of the clinical parameters were measured at baseline and at the 12-week follow-up for the study groups.

Clinical parameter	Gel group (*n* = 27)		Gel + PDT group (*n* = 27)		Comparison between the groups (*p*-value)
	0 Week	12 Weeks	0 Week	12 Weeks	
PD (mm) Mean ± SD Median (Q1, Q3)	5.57 ± 0.68 6 (5, 8)	4.12 ± 0.70 5 (4, 7)*⁣*^*∗*^	5.53 ± 0.72 6 (5, 8)	3.45 ± 0.89 4 (4, 6)*⁣*^*∗*^	1.000
CAL (mm) Mean ± SD Median (Q1, Q3)	5.51 ± 1.28 6 (5, 8)	4.59 ± 1.33 6 (4, 7)*⁣*^*∗*^	5.41 ± 1.28 6 (5, 8)	4.03 ± 1.04 4 (3, 5)*⁣*^*∗*^	0.035^#^
PI Median (Q1, Q3)	2 (1, 2)	1 (0, 1)*⁣*^*∗*^	2 (1, 2)	1 (0, 1)*⁣*^*∗*^	0.113
BOP (%) Median (Q1, Q3)	83 (75, 91)	40 (18, 75)*⁣*^*∗*^	85 (80, 88)	17 (10, 40)*⁣*^*∗*^	0.004^#^

*Note:* A comparison of clinical outcomes between the groups at week 0 and week 12 was conducted using the Wilcoxon signed-rank test on the most severe site of the selected tooth in each group, with measurements taken at six sites per tooth. Statistical significance (*⁣*^*∗*^ *p*  < 0.05) was assessed using the Wilcoxon signed-rank test for within-group comparisons at baseline, and the Mann–Whitney *U* test (^#^*p*  < 0.05) for between-group comparisons at 12 weeks post-treatment.

Abbreviations: BOP, bleeding on probing; CAL, clinical attachment level; PD, probing depth; PDT, photodynamic therapy; PI, plaque index.

## Data Availability

The data supporting the findings of this study are available within the article. Additional datasets generated during and/or analyzed during the current study are available from the corresponding author upon reasonable request.
